# Non-Invasive Monitoring of Inflammation in Inflammatory Bowel Disease Patients during Prolonged Exercise via Exhaled Breath Volatile Organic Compounds

**DOI:** 10.3390/metabo12030224

**Published:** 2022-03-03

**Authors:** Ben Henderson, Joris Meurs, Carlijn R. Lamers, Guilherme Lopes Batista, Dušan Materić, Carlo G. Bertinetto, Coen C. W. G. Bongers, Rupert Holzinger, Frans J. M. Harren, Jeroen J. Jansen, Maria T. E. Hopman, Simona M. Cristescu

**Affiliations:** 1 Department of Analytical Chemistry and Chemometrics, Institute for Molecules and Materials, Radboud University, 6525 AJ Nijmegen, The Netherlands; b.henderson@science.ru.nl (B.H.); joris.meurs@ru.nl (J.M.); guilhermelb@alumni.usp.br (G.L.B.); carlo.bertinetto@teijinaramid.com (C.G.B.); f.harren@science.ru.nl (F.J.M.H.); jj.jansen@science.ru.nl (J.J.J.); 2 Division of Human Nutrition and Health, Wageningen University & Research, 6708 WE Wageningen, The Netherlands; carlijn.lamers@wur.nl; 3 Department of Gastroenterology and Hepatology, Hospital Gelderse Vallei, 6716 RP Ede, The Netherlands; 4 Institute for Marine and Atmospheric Research, Utrecht University, 3584 CC Utrecht, The Netherlands; d.materic@uu.nl (D.M.); r.holzinger@uu.nl (R.H.); 5 Department of Physiology, Radboud Institute for Health Sciences, Radboud University Medical Center, 6525 EZ Nijmegen, The Netherlands; coen.bongers@radboudumc.nl (C.C.W.G.B.); maria.hopman@radboudumc.nl (M.T.E.H.)

**Keywords:** inflammatory bowel disease, exercise, breath analysis, PTR-ToF-MS, volatile organic compounds, butanoic acid

## Abstract

The aim of this study was to investigate volatile organic compounds (VOCs) in exhaled breath as possible non-invasive markers to monitor the inflammatory response in inflammatory bowel disease (IBD) patients as a result of repeated and prolonged moderate-intensity exercise. We included 18 IBD patients and 19 non-IBD individuals who each completed a 30, 40, or 50 km walking exercise over three consecutive days. Breath and blood samples were taken before the start of the exercise event and every day post-exercise to assess changes in the VOC profiles and cytokine concentrations. Proton transfer reaction time-of-flight mass spectrometry (PTR-ToF-MS) was used to measure exhaled breath VOCs. Multivariate analysis, particularly ANOVA-simultaneous component analysis (ASCA), was employed to extract relevant ions related to exercise and IBD. Prolonged exercise induces a similar response in breath butanoic acid and plasma cytokines for participants with or without IBD. Butanoic acid showed a significant correlation with the cytokine IL-6, indicating that butanoic acid could be a potential non-invasive marker for exercise-induced inflammation. The findings are relevant in monitoring personalized IBD management.

## 1. Introduction

Inflammatory bowel disease (IBD), including ulcerative colitis (UC) and Crohn’s disease (CD), is a condition that is characterized by chronic inflammation of the gastrointestinal tract [1]. Conventional drug-based treatment focuses on reducing inflammation, thereby relieving symptoms and inducing and maintaining long-term remission (absence of inflammation). However, many patients still experience symptoms such as fatigue despite being in remission with medication. Therefore, they look for supportive and adjunctive or even alternative therapies [2,3,4]. Exercise has been proposed as a treatment tool in patients suffering from IBD, with data suggesting that exercise may improve disease activity, quality of life, bone mineral density, and fatigue levels [5].

The general concern that exercise may exacerbate symptoms in IBD patients seems to depend on the amount and intensity of exercise. It was found that single bouts of high-intensity exercise can trigger an acute and transient exacerbation of inflammation [6]. It should be noted that the increase in inflammation markers directly after exercise is also valid for healthy individuals [7]. In the case of repetitive prolonged moderate-intensity exercise, the exercise-induced increase in the concentration of inflammation markers in healthy individuals gradually decreases after consecutive days of exercise, indicating an adaptive response [8]. Furthermore, it has been demonstrated that cytokine concentrations of IBD patients were comparable to non-IBD controls after prolonged moderate-intensity exercise (walking), indicating that this type of exercise can be performed safely without significant exacerbation of inflammation [9].

Exercise can also cause changes in the composition of the gut microbiota [10]. In particular, for IBD patients, exercise may have beneficial effects such as enriching the gut microbiota diversity and stimulating the proliferation of bacteria that modulate mucosal immunity and release of anti-inflammatory cytokines to regulate the environment in the gut lining and beyond. As a consequence, the cellular metabolism is altered; this is reflected in the production of specific metabolites measured as VOCs in breath, as demonstrated in a recent study in CD patients [11].

Breath volatile organic compounds (VOCs) are currently explored as non-invasive markers for various health conditions since they can provide a snapshot of a person’s metabolic profile at a given time [12,13]. Therefore, analysis of the VOCs produced during physiological and pathological processes (e.g., colon inflammation) by the microbiome may be useful in diagnosing and monitoring disease activity in IBD patients. These VOCs are released in the gut from which they subsequently enter the bloodstream and are further detected in exhaled breath via systemic circulation.

So far, a wide range of analytical techniques have been employed to measure the VOC profiles concerning IBD [14], including gas chromatography–ion mobility spectrometry (GC-IMS), ion-molecule reaction–mass spectrometry (IMR-MS), gas chromatography time-of-flight mass spectrometry (GC-TOF-MS), and selected ion flow tube mass spectrometry (SIFT-MS) [15,16,17,18]. In recent years, the primary focus of breath VOCs regarding IBD has been on diagnosis [16]. However, their potential use in monitoring the response of IBD patients to various therapies, such as exercise, has not been investigated so far.

This study aimed to explore breath VOCs as possible non-invasive markers for exercise-induced inflammation in IBD and non-IBD participants. For this, we assessed the response of both groups to repeated and prolonged moderate-intensity exercise by measuring changes in the VOC profiles using PTR-ToF-MS and their correlations with plasma cytokines.

## 2. Results

### 2.1. Identification of Breath VOCs Affected by Prolonged Exercise and IBD Condition

The results showed that breath VOC profiles of participants were affected by exercise and IBD condition (*p* < 0.001). Exercise accounted for 15% of the explained variance in the breath VOC profile, whilst the IBD condition contributed for 3%. The VOC concentrations were compared before and after exercise. Most breath VOCs increased in concentration in relation to exercise (*p* < 0.001). Only isoprene showed a decrease in concentration (*p* < 0.001), whilst dimethyl sulfide remained stable (*p* = 0.9884). The significant *m*/*z* values and their corresponding putative compound assignment (as specified in Section 4.5.2) are listed in Table 1 for both exercise and IBD condition. None of the significant ions were found to be affected by the walking distance (30, 40, or 50 km; *p* > 0.05).

### 2.2. Non-Invasive Assessment of Inflammation through Breath Analysis

**Figure 1:**
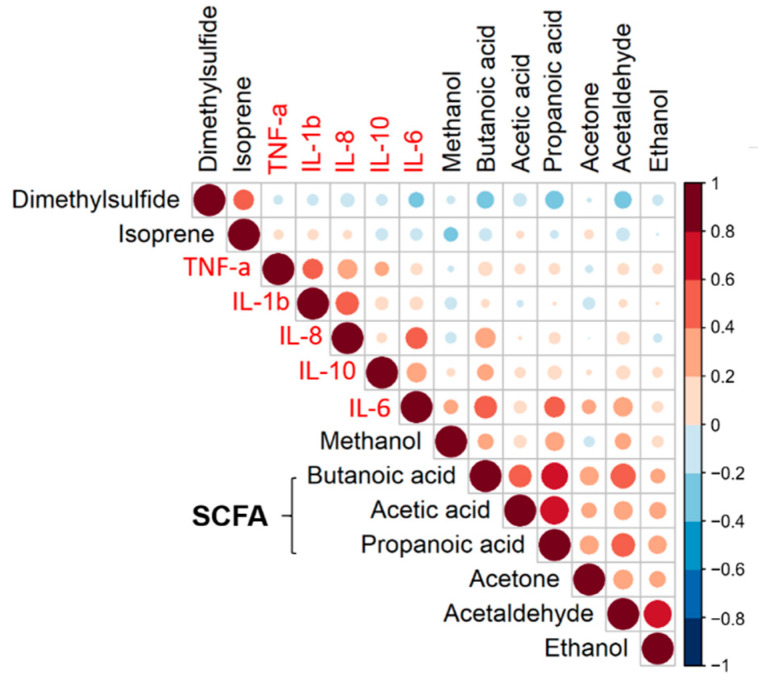
Correlation matrix for breath VOCs and inflammation markers ordered by hierarchical clustering (correlation distance function and ward linkage). Spearman rank correlations are represented by color intensity and circle size. Breath VOCs and cytokines are labeled black and red, respectively.

We further checked the relationship between the significant VOCs resulting from the ASCA model and the cytokines using Spearman rank correlation analysis. The results for all participants (non-IBD and IBD group) at all time points (day 0 to day 3) are displayed in Figure 1. The VOCs were ordered by hierarchical clustering (correlation distance function and ward linkage), revealing the short-chain fatty acid (SCFA) cluster represented by butanoic acid, acetic acid, and propanoic acid, respectively.

**Figure 2:**
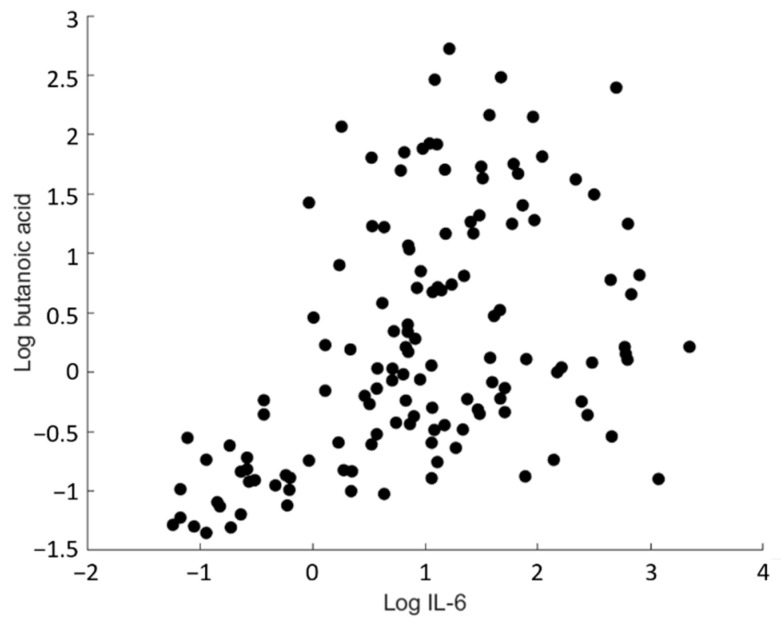
Log-log scatter plot of the butanoic acid concentration in exhaled breath of all participants for all time points (day 0–day 3) versus the plasma concentration of IL-6 measured at the same time points.

Butanoic acid and propanoic acid appear to correlate with IL-6. However, strict criteria were applied to obtain statistically and biologically meaningful correlations as outlined in Section 4.6.2. For a sample size of 37, the minimal correlation coefficient was found to be 0.445 or −0.445. Therefore, butanoic acid was the only compound to be significantly correlated with IL-6 (*r* = 0.5011, *p* < 0.001) (Figure 2).

**Figure 3:**
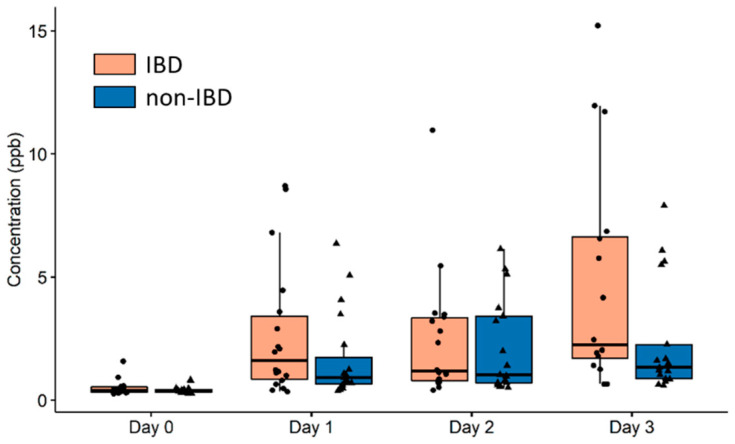
Box plots for the concentration of butanoic acid in breath for the IBD and non-IBD groups at each time point (Mann–Whitney test). There was no statistically significant difference between the two groups at any of the time points.

To investigate whether the correlation between butanoic acid and IL-6 was driven by the IBD or non-IBD group, butanoic acid concentrations were compared between both groups at each time point (Figure 3). No significant difference was found on any of the days.

## 3. Discussion

This study aimed to explore whether exercise-induced inflammation is reflected in the breath VOC profiles of IBD and non-IBD participants. Significant ions were identified using ASCA, which is a useful statistical tool to detangle minor (but significant) effects from larger ones. The results indicate that the breath VOC profile is primarily affected by exercise and that the IBD condition plays a minor role in comparison. However, it should be noted that the effect of the IBD condition is statistically significant (*p* < 0.001) and therefore also affects the breath VOC profile. The identified compounds were subsequently subjected to Spearman rank correlation analysis with cytokines. A significant correlation between butanoic acid and IL-6 was found (*r* = 0.5011; *p* < 0.001), indicating that butanoic acid may be used as a non-invasive marker for exercise-induced inflammation.

IL-6 is an interleukin that can act as both a pro-inflammatory cytokine and an anti-inflammatory myokine [19,20]. Lamers et al. observed that there was a strong response in IL-6 after the first day of walking compared to the baseline measurement. This implied a substantial increase in the production and secretion of myokines, which are primarily regulated by skeletal muscles. Therefore, this strongly suggests that the increase observed in IL-6 is directly related to the exercise and not to other potential factors, such as age and gender [9].

Using stringent criteria (*r* < −0.445 or *r* > 0.445 and *p* < 0.05), the correlation between butanoic acid and IL-6 was valid for all time points and groups. Butanoic acid is one of the three most common SCFAs produced by intestinal bacteria via anaerobic fermentation of non-digestible dietary fibers [18]. This metabolite plays an important role in the maintenance of intestinal homeostasis through anti-inflammatory actions [21,22,23]. Propanoic acid also showed a significant correlation with IL-6 (*r* = 0.4178; *p* < 0.001); however, the Spearman rank correlation was just below the set threshold.

Furthermore, exercise is associated with an increased plasma IL-6 concentration from muscles which also has an anti-inflammatory effect by increasing glucagon-like peptide-1 secretion from intestinal L cells and pancreatic alpha cells [24]; hence, the positive correlation between butanoic acid and IL-6.

We observed no difference in the butanoic acid concentrations of the IBD versus the non-IBD group measured at all time points (Figure 3). This suggests that the butanoic acid concentration may act as a general non-invasive marker for exercise-induced inflammation. Our results are in line with previous findings in the same population by Lamers et al. who found no difference between IBD and non-IBD in cytokine concentration suggesting that this type of exercise can be safely performed by IBD patients [9].

The results of our study can be broadly used since we measured breath volatiles and inflammation markers from both CD and UC patients. No difference in breath butanoic acid levels was found between CD and UC patients at any of the time points (*p* > 0.05). However, the study has some limitations that should be considered. First, no restrictions were placed on the participants, thus limiting the investigation of the potential influence of exogenous factors, such as diet. However, the VOCs identified during this study have been previously identified in similar studies regarding repeated and prolonged exercise or IBD condition [17,18,25,26]. Therefore, we have confidence that the changes observed in these VOCs over time are primarily due to the effect of exercise or IBD condition rather than the effect of diet or other exogenous factors. Secondly, 11 participants in the IBD group were using medication for their condition. As a consequence of the limited sample size, the effect of the IBD-related medication on the breath VOC profile could not be investigated. Finally, the breath SCFAs were measured at the systemic level without accounting for other sources (e.g., liver). Nevertheless, it has been previously shown that such contributions to breath are much lower than of the intestine [27].

## 4. Materials and Methods

### 4.1. Subjects

In this study, we included 37 participants enrolled in a multiple-day walking event, in which they walked 30, 40, or 50 km a day at a self-selected pace for 3 consecutive days (Table 2). The participants were comprised of 18 IBD and 19 non-IBD individuals matched for age (±5 years) and gender. The inclusion criteria for the IBD participants were that they must be 18 years of age or older and be diagnosed with CD or UC by a gastroenterologist. IBD participants were excluded if using specific biologicals (such as infliximab, adalimumab, golimumab, ustekinumab) that could reduce cytokine concentrations. Non-IBD participants were 18 years of age or older with no history of IBD or other gastrointestinal diseases. None of the participants had a reported respiratory disease. No restrictions on food or drink intake were placed on participants. The study from which the participants were recruited was approved by the Medical Ethical Committee (MEC) region Arnhem-Nijmegen (CMO registration number: 2019-5375). All participants provided written informed consent. This study was conducted in accordance with the Declaration of Helsinki and was registered at Trialregister.nl as NL7872. The sample size was limited to a maximum of 20 participants per group by the MEC.

### 4.2. Exhaled Breath Sampling Protocol

Breath samples were collected from the participants in duplicate at the following time points: pre-walking split over two days prior to the start of the event (day 0) and post-walking on day 1 to day 3. The sampling was performed with a small user-friendly custom-built breath sampler that allows collection of the end-tidal portion of the exhaled breath. The procedure is described in detail elsewhere [28]. Briefly, the participants were asked to exhale via a mouthpiece bacterial filter (GVS, Morecambe, UK) through a one-way breathing tube fitted with Teflon tubing connected to a 3 L Tedlar^®^ bag. A small discard bag of 150 mL was attached to the start of the sampling line device to collect the dead space portion of the exhaled breath (not for analysis). Participants were asked to provide a single exhalation per bag (typically 1.5 L), at an exhalation rate of 100 mL/s.

### 4.3. PTR-ToF-MS Instrumental Set-Up

The breath samples were measured with a PTR-ToF-MS instrument (model PTR-ToF8000, Ionicon Analytik GmbH, Innsbruck, Austria). The principle of PTR-ToF-MS has been described in detail elsewhere [29,30]. Briefly, breath VOCs react with hydronium (H_3_O^+^) ions in the drift tube section of the instrument. A soft chemical ionization of VOCs takes place by means of proton transfer in case the proton affinity of the VOC is greater than that of water (691 kJ·mol^−1^). Ions are then separated by their time of flight and subsequently detected.

The content of the Tedlar^®^ bags was measured within 2 h from the sampling. Prior to analysis, the Tedlar^®^ bag was placed in an incubator at 37 °C for 10 min and was subsequently attached to the inlet of the PTR-ToF-MS. The breath samples were measured continuously for a minimum of 2 min. A selection of ions was monitored in real time to ensure a stable signal was achieved. The exhaled breath composition was sampled at a flow of 24 mL/min via a 2 m long heated (80 °C) ¼″ Silcosteel^®^ transfer line. The drift tube temperature and voltage were set to 80 °C and 480 V, respectively, with an operating pressure of 2.5 mbar, resulting in a reduced electric field (E/N) of ~120 Td. Before each measurement, selected ions were monitored until a stable baseline was reached. Breath sampling repeatability was checked using Lin’s concordance correlation coefficient [25].

### 4.4. Blood Inflammation Markers

The inflammation markers included for correlation with relevant VOCs were IL-6, IL-8, IL-10, IL-1β, and TNF-α. Details of their sample collection and analysis procedure can be found elsewhere [9].

### 4.5. Data Processing

#### 4.5.1. Spectral Processing

PTR-ToF-MS data were processed using the PTRwid software [31]. The final mol ratio values were corrected for the transmission efficiency deduced from a calibration experiment as reported earlier [32]. The average and merge function of PTRwid was used to average the data to 5 s time resolution.

#### 4.5.2. Compound Identification

The instrument allows the putative assignment of VOCs by retrieving the molecular formula for each ion. To further assist in the identification, next to reported literature on breath research [25,33], Pearson’s correlation coefficient (r) was calculated to identify related fragments or clusters. An *r* > 0.85 was considered for an ion to originate from the putatively assigned parent ion. The identification of the SCFAs was confirmed with the use of standards. As such, compound names have been used throughout rather than *m*/*z* values alone.

The parent ion of butanoic acid (*m*/*z* 89.06) could not be identified unequivocally due to interference arising from the ^13^C isotope of dimethylacetamide (*m*/*z* 88.12), which is a known contaminant found in Tedlar^®^ bags [34]. Therefore, the fragment ion *m*/*z* 71.05 was used to quantify butanoic acid.

### 4.6. Statistical Analysis

#### 4.6.1. ANOVA Simultaneous Component Analysis (ASCA) for Identification of Significant Ions in the Breath VOC Profile

ANOVA simultaneous component analysis (ASCA) [35], a multivariate version of ANOVA to analyze datasets with an underlying experimental design, was used to disentangle the effect of exercise from that of IBD on the breath profile for the PTR-ToF-MS data. Prior to ASCA, the breath VOC data were transformed using the inverse hyperbolic sine (arcsinh). This method first decomposes the data matrix X into effect matrices corresponding for each factor or interaction (Equation (1)):

(1) $$X=X_{m}+X_{D}+X_{IBD}+X_{i}+X_{e}\;$$

where *X_m_* contains the overall means of each *m*/*z*, *X_D_* is the effect matrix of the day of exercise, *X_IBD_* that of the IBD group, *X_i_* that of the individual (nested in the IBD group), and *X_e_* contains the residuals. These effect matrices are obtained by applying ANOVA, with type III corrections for unbalanced data [36], to every *m*/*z* separately and then recollecting all obtained columns. After this decomposition, PCA is applied to each effect matrix to highlight the specific patterns and relationships among *m*/*z* that are correlated to each effect. This method allows for a straightforward interpretation of the variation induced by the different factors of the experimental design. The amount of variance explained by each effect is computed from the sums-of-squares of the corresponding matrix. The validation is performed through permutation tests and by comparing the sums-of-squares of the effect matrices of random models with those of the real model. The ions that were important for the relevant effect were selected based on their leverage value (in essence: the squared PCA loadings) using a 95% confidence threshold as reported in Nueda et al. [37].

#### 4.6.2. Statistical and Correlation Analysis of Significant VOCs with Plasma Cytokines

Assessment of correlation between plasma cytokines [9] and breath VOCs was performed using Spearman rank correlation coefficient. The minimum Spearman’s rank correlation coefficient for the given sample size was calculated according to Equation (2), where *N* is the sample size, *Z_α_* the z-score for the selected *α*, *Z*_1−*β*_ the z-score for the selected power, and *C* the Fisher transformation of the correlation coefficient (*r*) (Equation (3)) [38]. The values for *α* and *β* were set at 0.05 and 0.80, respectively.

(2) $$N={\left(\frac{Z_{\alpha }+Z_{1-\beta }}{C}\right)}^{2}+3$$

(3) $$C=\frac{1}{2}\ln\left(\frac{1+r}{1-r}\right)\;\;$$

Comparison of breath VOC concentrations between IBD and non-IBD participants for each time point was conducted using the Mann–Whitney test. For comparison between more than two time points and/or groups, data were log-transformed and subjected to ANOVA. Correction of *p*-values for multiple comparisons was performed according to the Benjamini–Hochberg procedure [39]. Corrected *p*-values less than 0.05 were considered significant.

## 5. Conclusions

Taken together, this study provides evidence for the potential role of SCFAs, specifically butanoic acid in exhaled breath as a non-invasive marker for exercise-induced inflammation. Repeated prolonged moderate-intensity exercise affects exhaled butanoic acid and IL-6 of participants with or without IBD in the same manner. Due to the direct connection of SCFAs to the gut microbiota, our findings can be used in future research to establish the planning of exercise regimes and to monitor the progress of many inflammatory diseases, in particular for IBD patients.

## Figures and Tables

**Table 1:** Significant ions in relation to exercise and IBD condition as derived from ASCA and their putative identity. The *m*/*z* values given in parentheses indicate product ion fragments or hydrate clusters.

*m*/*z*	Product Ion Formula	Assigned Compound
33.03	CH_4_OH^+^	Methanol
45.03	C_2_H_4_OH^+^	Acetaldehyde
47.05	C_2_H_6_OH^+^	Ethanol
59.05	C_3_H_6_OH^+^	Acetone
61.03 (43.02, 79.05)	C_2_H_4_O_2_H^+^ (C_2_H_2_OH^+^, C_2_H_6_O_3_H^+^)	Acetic acid
63.03	C_2_H_6_SH^+^	Dimethyl sulfide
69.07 (41.04)	C_5_H_8_H^+^ (C_3_H_4_H^+^)	Isoprene
(71.05)	C_4_H_6_OH^+^	Butanoic acid
75.04 (57.04, 93.05)	C_3_H_6_O_2_H^+^ (C_3_H_4_OH^+^, C_3_H_8_O_3_H^+^)	Propanoic acid

**Table 2:** Demographic data of the study participants.

Characteristic	IBD (*n* = 18)	Non-IBD (*n* = 19)
Age (years)	54 ± 11 ^a^	54 ± 14 ^a^
Gender (F/M)	11/7	11/8
Walking distance per day (30/40/50 km)	5/11/2	2/13/4
Body mass index (kg/m^2^)	25.7 ± 3.8 ^a^	26.0 ± 4.5 ^a^
IBD medication use (yes/no)	11/7	-
Smoking status (active smoker/non-smoker/ex-smoker)	1/9/8	0/13/6

^a^: Data are presented as the mean ± standard deviation.

## Data Availability

The data presented in this study are available on request from the corresponding author. The data are not publicly available due to the institutional policy regarding data sharing.
